# Acute Confusional Migraine: Case Reports and Discussion as a Distinct Entity

**DOI:** 10.1155/crnm/5382669

**Published:** 2025-08-20

**Authors:** Karen dos Santos Ferreira, Ana Miriam Velly

**Affiliations:** ^1^Headache Clinic, Montreal Neurological Clinic, Montreal, Quebec, Canada; ^2^Suroit Hospital, Salaberry-de-Valleyfield, Quebec, Canada; ^3^Faculty of Dentistry, Jewish General Hospital, McGill University, Montreal, Quebec, Canada

**Keywords:** acute confusional migraine, aura, confusion, migraine

## Abstract

**Background:** Acute confusional migraine (ACM) is a migraine variant manifesting with confusion, agitation, disorientation, altered mental status, and/or memory deficits. ACM has notably been excluded from the International Classification of Headache Disorders (ICHD-3 β), despite previous literature describing 120 cases and proposing a standardized classification. Considering these findings, clinicians must be aware of this condition as it can be confounded with other serious health conditions (e.g., stroke, encephalitis, and epilepsy).

**Objective:** Herein, we describe three cases with altered consciousness during a migraine attack, discussing diagnostic criteria, treatment, prognostic implications, and future perspectives.

**Results:** The first case, an 18-year-old male, presented to the emergency room with migraine with visual aura, followed by confusion and torpor. After 24 h, he was completely recovered without deficits. In the second case, a 30-year-old woman in puerperium presented with a visual aura followed by headache, confusion, and disorientation. She recovered her conscience without deficits. The third patient, a 36-year-old woman, showed up in the emergency room presenting migraine, hemiplegia, and confusion. She recovered without deficits after 8 days. Finally, the genetic panel confirmed familial hemiplegic migraine (for the third patient). All the tests, including brain computed tomography (CT) scan, angioscan, brain magnetic resonance imaging (MRI), lumbar puncture (LP), and toxicological workup, had normal results for all three patients. They were treated for migraine with long-term control.

**Conclusion:** ACM is a significant condition that can be mistaken for other serious health issues. Health professionals need to be better informed about their diagnosis and management strategies. Therefore, we proposed criteria to include ACM in the ICHD-3 β classification, and we emphasize the need for future studies to improve understanding and treatment of this condition.

## 1. Introduction

Migraine is one of the most common disorders worldwide, resulting in significant social costs, loss of work hours, and expensive treatments [1, 2]. In 2016, the global age-standardized prevalence of migraine was 14.4% [1].

According to the International Classification of Headache Disorders (ICHD-3 β), migraine is characterized by moderate to severe pain intensity, usually unilateral, lasting from 4 to 72 h, in addition to other associated symptoms such as nausea, vomiting, sensitivity to light, sound, and transitory symptoms called auras [3]. The aura can lead to visual, language, motor, and sensory disturbances as well as brainstem symptoms such as dysarthria, vertigo, ataxia, and decreased loss of consciousness [3].

The pathophysiology of migraine involves neurogenic inflammation and vascular changes, characterized by recurrent activation and sensitization of the trigeminal-vascular pathways in individuals with a genetic predisposition for increased brain excitability [4]. The cortex involvement in migraine is well established during the aura phase, which is associated with a phenomenon called cortical spreading depolarization, a slowly spreading wave of neuronal and glial depolarization, followed by a suppression of activity [5, 6].

In this context, a migraine variant, called acute confusional migraine (ACM), has been identified, manifesting with acute confusion, agitation, disorientation, altered mental status, speech difficulties, and memory deficits. Ehyai et al. first described ACM in 1978 [7]. In addition, it may be the first presentation of migraine in children and/or adolescents. Previous literature (systematic review) searching for “confusional state in migraine” and “confusional migraine” included a total of 120 cases and proposed a standardized classification [8].

To support the basis of this presentation, changes in EEG readings have been noted, particularly during prolonged agitation and mental confusion associated with headache attacks, especially in adolescents [9]. The ictal EEG may display diffuse slow abnormalities and a specific pattern known as frontal intermittent rhythmic delta activity (FIRDA) [9].

Although it is not a part of the ICHD-3β, it represents a significant clinical syndrome that can lead to high levels of disability, emergency room visits, and diagnostic uncertainty [8]. The literature regarding this variant is limited, likely due to its exclusion from the ICHD-3β. It remains unclear whether ACM is a manifestation of migraine biology reflecting various phases or a distinct clinical entity.

Considering all of this, the primary objective of this paper is to describe three cases of confusional migraine and discuss its pathophysiology in the context of migraine phases. The secondary objective is to inform general physicians about this atypical presentation of migraine, which could lead to misdiagnosis and inappropriate treatments.

## 2. Case Reports

### 2.1. Patient 1

An 18-year-old male presented to the emergency room with a right-sided visual scotoma, followed by a severe pulsatile headache, right-sided paresthesia, vertigo, and confusion (see Table 1). After 1 h, he deteriorated into a state of torpor, prompting the physicians to transfer him to the intensive care unit and intubate him, due to reduced consciousness threatening the airway. He had been presenting migraine with visual aura for the past 2 years, without specific treatment. Otherwise, his past medical history was unremarkable, and his family history was negative for migraine. He did not describe drug or alcohol consumption. An extensive investigation was conducted, including a brain computed tomography (CT) scan and angioscan, brain magnetic resonance imaging (MRI), lumbar puncture (LP), and toxicological workup (all of them with normal results). The EEG revealed polymorphic center-temporal activity without epileptic discharges. After 24 h, he recovered without deficits, allowing the medical team to extubate him. The preliminary hypothesis included ACM, migraine with complex basilar aura, and headache with neurological deficits and cerebral spinal fluid lymphocytosis (HANDL syndrome). HANDL syndrome was excluded after a normal LP. He was treated (as per basilar migraine) with verapamil, but he experienced intolerance in the following weeks, leading to a switch in medication to lamotrigine. Over 1 year, he did not experience any recurrence of aura or confusion, although he did continue to have some migraines without aura. Finally, he decided to stop treatment without medical advice, and he had another migraine with aura (paresthesia and confusion) lasting about 2 hours. We recommended keeping the treatment for the long term.

### 2.2. Patient 2

A 30-year-old woman arrived at the emergency room due to an unusually severe headache. Two hours prior to her arrival, she experienced a visual aura (characterized by zig-zag patterns right-sided), which was followed by a pulsatile headache and confusion and disorientation (see Table 1). Her physical examination, vital signs, and blood pressure were normal. Two months before this incident, she had given birth without complications, and she was breastfeeding, which contributed to sleep deprivation. Considering her important confusional state, her family was concerned about the possibility of a stroke and her ability to take care of her baby, prompting them to seek medical help. Her past medical history included migraines with visual aura for the last 10 years without specific treatment, and her family history was negative for migraines. She did not have addictive behavior or alcohol consumption. Investigations were conducted, including brain CT scan and angioscan, brain MRI, LP, and toxicological workup, all with normal results. An EEG showed slow abnormalities in anterior regions without epileptic discharges. After 6 h, she was conscious and oriented again, with amnesia related to the last hours leading up to her arrival. The preliminary hypothesis included ACM and migraine with complex aura (probably triggered by sleep deprivation and postpartum hormonal changes). She was treated with metoprolol, which provided excellent control of her migraine. After 14 months of follow-up, she did not have any recurrence of aura or confusion (experiencing only mild migraine without aura).

### 2.3. Patient 3

A 36-year-old woman presented to the emergency room with localized frontal pain, paralysis on the left side of her body, blurred vision, and delusions (see Table 1). Due to her confusion and hemiplegia, she was investigated for an acute stroke and subsequently transferred to the intensive care unit. All tests, including brain CT scan and angioscan, brain MRI, LP, and toxicological workup, returned with normal results. The EEG showed slow abnormalities without epileptic discharges. She experienced confusion and weakness on her left side for 8 days, recovering without deficits. Since she has been presenting migraines for 30 years without specific treatment, and her mother had a diagnosis of hemiplegic migraine, she was referred to a genetic clinic. Finally, the migraine panel detected a heterozygous pathogenic variant in ATP1A2 c.901G > C, p. (Gly301Arg), confirming a diagnosis of familial hemiplegic migraine (FHM). She was initially treated with acetazolamide. However, her migraines remained recurrent without hemiplegia and without confusion. Lamotrigine was added, which effectively controlled her migraines, and she did not experience any recurrence of confusion over the following 48 months.

## 3. Discussion

In this report, we describe three patients who experienced altered consciousness during migraine attacks. Each patient underwent extensive investigation, and no alternative diagnoses were found to explain their clinical presentations. It is important to empathize that this atypical presentation could lead to misdiagnosis (i.e. stroke and epilepsy) and inappropriate treatment, as IV thrombolysis or antiepileptic drugs, increasing the risk of morbidity and mortality.

The mechanisms underlying the complex relationship between migraines, cognitive changes, and consciousness remain poorly understood, despite examples in the literature. Farooqi et al. performed a systematic review and described 120 patients presenting confusional symptoms during a migraine attack [8]. Although the exact pathogenesis connecting migraines with confusional symptoms is not fully elucidated, several theories exist.

First, the model of migraine is an interesting example where the involvement of the cortex is well established, notably through the underlying phenomenon of cortical spreading depolarization (a cortical wave of neuronal and glial depolarization followed by activity drop) [5]. Moreover, migraines have been linked to cognitive deficits in different contexts, such as being unable to think or concentrate and processing working memory and executive function activities (such as working or taking care of children). This can be related to cortical spreading depolarization [6]. There is a direct relationship between attention, transient information retention, memory, and the activity of neurons in the prefrontal cortex and limbic system (which may be impaired during migraine attacks). Patients commonly report attention and cognitive disturbances, even when awareness is generally intact. Attack-related cognitive symptoms can be intense and disabling [10, 11].

Second, changes in EEG patterns have been observed during headache attacks, particularly among adolescents, which may manifest as prolonged agitation and mental confusion. Ictal EEG often shows diffuse slow abnormalities, along with a distinct pattern known as FIRDA [9]. Prolonged EEG could increase diagnostic yield, particularly in distinguishing epileptic from nonepileptic confusional states. However, in most clinical settings, the feasibility for patients in emergency or acute care contexts remains complicated. It could not be readily accessible.

Third, an activation of the brainstem during a migraine attack (especially in basilar migraine) can also be implicated in the pathogenesis of ACM. Episodes of altered consciousness have been seen in FHM, basilar migraine, and cerebral autosomal dominant arteriopathy with subcortical infarcts (CADASIL). It is considered a reversible encephalopathy, generally following a migraine with or without aura. In up to 10% of CADASIL patients, a reversible encephalopathy is the first presentation leading to diagnosis. The strong association with migraine suggests a shared pathogenesis [12].

Furthermore, FHM and CADASIL share genetic abnormalities located on Chromosome 19, which can impact ion channel function and vascular smooth muscle cells. In some patients with these channelopathies, it has been speculated that channel dysfunction could promote a trigger for cortical spreading depression leading to brain cellular damage and blood–brain barrier disruption after head trauma or even stressful situations [13].

In summary, despite the evidence of this clinical syndrome and the disability implicated (including visits to the emergency room and hospitalization), there are no consensus classification criteria for ACM. The exclusion from the ICHD-3 β may result in a decreased interest in publishing these cases. Nevertheless, a standardized classification criterion (to be included in the ICHD-3 β appendix) was proposed by Farooqi et al. In 2018 (see Table 2) [8]. It would include this subtype of migraine in the A1.6. Episodic Syndromes that may be Associated with Migraine. The Appendix is supposed to include novel headache entities that have not been sufficiently validated by the research conducted so far. Although the ICHD-3 β also includes an item related to a decreased level of consciousness (Glasgow Coma Scale ≤ 13) in the 1.2.2 Migraine with Brainstem Aura, it might be more suitable to classify this syndrome as an episodic syndrome since it can occur independently of brainstem aura [3]. In addition, EEG findings can indicate cortical involvement, not solely brainstem activity [9].

Overall, we found the first criterion proposed by Farooqi et al. in 2018 to be relevant. However, we recommended some minor adjustments, specifically regarding the exclusion of combative behavior, which could lead to misclassification bias related to drug or alcohol abuse. In addition, we suggested including normal brain imaging, such as a brain CT scan or MRI, as part of the criteria (see Table 2).

Regarding treatment, there is no established consensus for managing these patients, and the duration of treatment remains unclear. Given the complexity of the presentation and the frequent involvement of aura, it seems reasonable to administer preventive treatment for migraines (similar to that for migraines with or without aura) for an extended period. In this case report, we utilized metoprolol, verapamil, diamox, and lamotrigine, which successfully controlled the symptoms.

## 4. Limitations of This Study

The proposed diagnosis of ACM is based on a first episode, presumptive, in the presence of clinical symptoms, temporal relation with a migraine attack, and careful exclusion of other causes. Waiting for a second episode before making a diagnosis could increase accuracy, although it may not be appropriate or practical in real-world clinical settings. Many patients may never experience a second attack. As seen in other transient neurological syndromes such as transient global amnesia (TGA) or reversible cerebral vasoconstriction syndrome (RCVS), a single, well-characterized event with a compatible clinical and investigational profile may be sufficient for diagnosis if supported by careful exclusion of other causes.

In addition, long-term EEG could have further strengthened the exclusion of nonconvulsive status epilepticus. However, in most clinical settings, 24-h video EEG is not readily accessible, especially for patients in emergency or acute care contexts. It typically requires referral, prolonged wait times, and inpatient monitoring, which may prolong hospitalization, increase patient distress, and delay diagnosis and treatment.

Finally, all our patients underwent brain MRI, including diffusion-weighted imaging, but perfusion imaging was not performed. Perfusion CT may provide valuable insight into the assessment of confusional migraine (excluding other diagnostics such as PRES or ischemic changes) and support the hypothesis. We have added this test to the diagnostic criteria proposed for future cases and studies, particularly in evaluating altered consciousness during a migraine with aura attack.

## 5. Future Perspectives

We should encourage the publication of more studies in the future to support the inclusion of this syndrome in the ICHD-3 β classification.

## 6. Conclusion

ACM is a significant condition that can be mistaken for other serious health issues. Health professionals need to be better informed about their diagnosis and management strategies. Therefore, ACM should be included in the ICHD-3 β classification, and future studies are necessary to further understand and address this condition.

### 6.1. Learning Points

The key conclusions from this study can be summarized as follows:

1. ACM is a migraine variant manifesting with confusion, agitation, disorientation, altered mental status, and/or memory deficits.
2. ACM can be mistaken for other serious health issues (e.g., stroke, encephalitis, and epilepsy).
3. An active search for other diseases is recommended for all patients experiencing migraine, followed by a decreased level of consciousness.
4. Further research is needed to gain a better understanding of this condition, which could facilitate its inclusion in the ICHD-3 β classification.
5. It seems reasonable to treat this migraine variant with preventive treatment (as per migraine with or without aura) over an extended period.

## Figures and Tables

**Table 1:** Case reports related to acute confusional migraine.

Patient gender	Age	Presentation	Diagnostic	Duration confusion	Headache onset	Treatment inpatient	Treatment outpatient	Outcome
Male	18	Headache, visual aura, paresthesia, vertigo, confusion + torpor	Migraine with aura	24 h	Before confusion	Metoclopramide, Tylenol, dexamethasone	Verapamil (intolerance), lamotrigine	Follow-up 18 months recurrence, after stopping medication
Female	30	Headache visual aura, confusion	Migraine with aura	6 h	Before confusion	Metoclopramide, Tylenol	Metoprolol	Follow-up 14 months, no recurrence
Female	36	Headache, hemiparesis, confusion	Familial hemiplegic migraine	8 days	Before confusion	Dexamethasone, acetazolamide	Acetazolamide, lamotrigine	Follow-up 48 months, no recurrence

**Table 2:** Diagnostic criteria for acute confusional migraine, proposed for International Classification of Headache Disorders (ICHD-3 β).

Criteria proposed in 2018 by Farooqi et al.	Criteria proposed by Ferreira et al.
A. At least one attack, fulfilling criteria B to G, not attributed to other medical disorder and/or drug intoxication	A. At least one attack, fulfilling criteria B to G, not attributed to other medical disorder and/or drug intoxication

B. At least one of the following: 1. Decreased attention 2. Altered awareness 3. Impaired cognition (disorientation and/or deficits in attention, executive function, and memory)	B. At least one of the following: 1. Decreased attention or disorientation 2. Altered awareness

C. At least one of the following: 1. Agitation or combative behavior 2. Perception disturbances (i.e., visuospatial abnormalities and photophobia) 3. Slowing or frontal intermittent rhythmic delta activity on EEG with complete resolution within a week 4. Aura (reversible visual, sensory, language, or brainstem disturbance) for < 1 h (typical)	C. At least one of the following: 1. Typical aura (reversible visual, sensory, language, or brainstem disturbance) for < 1 h 2. Slowing or frontal intermittent rhythmic delta activity on EEG with complete resolution within a week 3. Perception disturbances (i.e., visuospatial abnormalities)

D. Complete resolution within 24 h or after sleep with partial or complete amnesia of the event	D. Complete resolution within 24 h or after sleep with partial or complete amnesia of the event

E. Normal neurological or no persistent neurologic deficit examination following the attack	E. Normal neurological examination and brain image (i.e., brain CT scan with perfusion or MRI)

F. At least one of the following: 1. Past medical history of migraine 2. Family history of migraine 3. Headache, if present, may occur before, during, and after the confusional state	F. At least one of the following: 1. Past medical history of migraine 2. Family history of migraine 3. Headache with migraine features occurring before, during, or after the confusional state

G. Not attributed to another disorder	G. Not attributed to another disorder

## Data Availability

The authors declare that the data supporting the findings of this study are available within the article.
